# Supercritical CO_2_ drying of alginate/zinc hydrogels: a green and facile route to prepare ZnO foam structures and ZnO nanoparticles†

**DOI:** 10.1039/c8ra02129e

**Published:** 2018-06-06

**Authors:** Hicham Abou Oualid, Othmane Amadine, Younes Essamlali, Karim Dânoun, Mohamed Zahouily

**Affiliations:** Laboratoire de Matériaux, Catalyse & Valorisation des Ressources Naturelles, URAC 24, Faculté des Sciences et Techniques, Université Hassan II Casablanca B.P. 146 20650 Morocco m.zahouily@mascir.com; MAScIR Foundation, VARENA Center, Rabat Design Rue Mohamed El Jazouli, Madinat Al Irfane 10100 Rabat Morocco

## Abstract

In the present study, we investigate a simple and effective synthetic protocol to produce zinc oxide foams by a facile solution-based method using alginate gelation. The influences of the zinc concentration and the drying process on the structural, textural and morphological properties of the synthesized ZnO nanomaterial were studied and discussed. The components of these nanomaterials were characterized by several techniques to demonstrate the effectiveness of the adopted synthetic route in controlling the growth of the ZnO nanoparticles. XRD analysis revealed that the as-prepared ZnO nanomaterial crystallizes in the hexagonal wurtzite structure. The room temperature photoluminescence (PL) spectra of ZnO show ultra-violet (UV) and visible emissions. SEM analysis revealed the porous texture of the prepared zinc oxide. TEM analysis confirmed the nano dimensions of the synthesized zinc oxide nanoparticles. A comparative study of conventional air drying *versus* supercritical drying was conducted to determine the influence of each mode of drying on the structural, textural and morphological as well as optical properties of the synthesized ZnO nanoparticles.

## Abstract 2

In the present study, we investigate a simple and effective synthetic protocol to produce zinc oxide foams by a facile solution-based method using alginate gelation.

## Graphical abstract

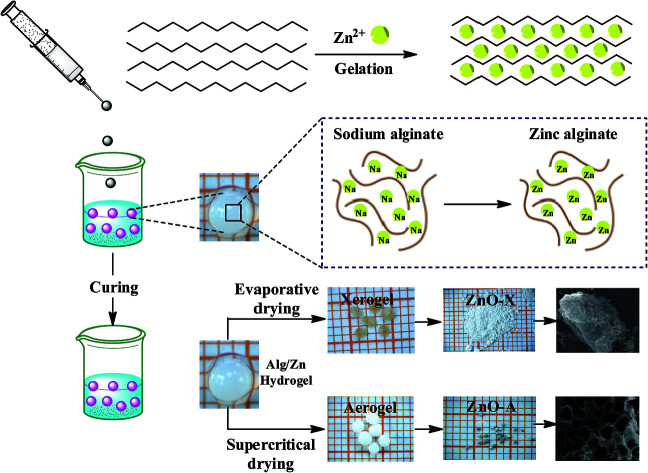

## 1. Introduction

Zinc oxide is one of the most important oxides, with very interesting and unique physical and chemical properties.^1,2^ This has resulted in its numerous applications in cutting-edge technology sectors, for example, sensors,^3–5^ catalysis & photocatalysis,^6–8^ solar cells & photovoltaic devices,^9–11^ optoelectronic devices,^12–14^ biomedicine,^15–17^ light-emitting diodes^18,19^ and other diverse areas.^20–23^ However, the specific properties of this oxide are influenced by several parameters including the structure, size, shape, morphology, and the porous texture. Several methods have already been developed to synthesise ZnO (powder or thin films), especially to control its morphology and porous texture, mainly by the following processes: chemical vapor deposition,^24^ radio frequency magnetron sputtering,^25^ spray pyrolysis,^26^ molecular beam epitaxy,^27^ pulse laser deposition,^28^ atomic layer deposition,^29^ hybrid-vapor phase epitaxy,^30^ metal–organic vapor phase epitaxy,^31^ vapor-phase synthesis,^32^ synthesis from the melt,^33^ hydrothermal synthesis,^34^ precipitation,^35^ co-precipitation,^36^ and sol–gel^37^ and sol–gel assisted ultrasonic methods.^38^ In line with this, several morphological forms of zinc oxide including nanorods, nanotubes, hollow structures, nanocubes, flower-like structures, and nanospheres were prepared.^39^ Ungula *et al.* have synthesized ZnO nanoparticles *via* a sol–gel mineralization process using a sol–gel method with a systematic characterization by several analytical techniques to investigate the effect of the volume ratio of water *versus* ethanol on the structural, morphological and optical properties of ZnO nanoparticles.^40^ Furthermore, research has shown that organizing nanoparticles into a hierarchically porous architecture with interconnected pores and a high specific surface area optimizes the functional performance and even renders new functionalities. Furthermore, several natural products have been exploited to develop nanostructured materials such as nanoparticles,^41,42^ nanostructured oxides^43,44^ and nanocomposites^45^ by controlling their nucleation and growth. In the case of ZnO, several examples were published in recent years mentioning the use of templates derived from renewable raw materials.^46–49^ Indeed, Carp *et al.* have succeeded in developing a model approach for the preparation of ZnO hollow spheres and ZnO/C composites using carbonaceous spheres derived from starch as templates.^50^ Various saccharides (mono-, di-, or polysaccharides) were also tested in the synthesis of different ZnO-composite architectures like rod, spindle, and solid and hollow spherical-like structures.^51^ A further hydrolysis process of zinc acetyl acetonate in 1,4-butanediol was applied to afford nanostructured ZnO with versatile morphologies and optical properties.^52^ The authors indicate that the reaction conditions (temperature, time and zinc source concentration) influence the nanocrystallite sizes (from 8.1 to 13.2 nm), the sphere diameters (ranging from 50 up to 250 nm), the internal structures of the spherical aggregates (hollow or solid) and their specific surface areas (from 31 to 92 m^2^ g^−1^). However, for this type of process, little attention has been given to the use of sodium alginate as a template to synthesise ZnO.^53,54^ Thirumavalavan *et al.* have used corn and sodium to prepare nanostructured ZnO with various morphologies and crystalline sizes.^55^ The method of synthesis consists of a one-pot mixture of two solutions: one containing zinc cations and the second the gel of the alginate, the starch or the modified starch.

Additionally, the structure and porosity of the synthesized materials, and therefore all of their physicochemical properties, depend directly on their method of synthesis and especially their drying process.^56^ This step consists of extracting the solvent contained in the pores of the hydrogel by trying to best preserve the structure and integrity of the solid network formed during the material growth step. Different drying methods are possible.^57^ The main drying methods are the conventional evaporation process of the interstitial solvent, which gives rise to xerogels, or supercritical drying which leads to aerogels. The first method of drying is the most used, but it suffers from various disadvantages. It causes densifying and cracking of the dry gel, due to the presence of a liquid–gas interface, which produces the appearance of a high surface tension (capillarity) effect on the solid network. To overcome the constraints of the capillary pressure during conventional drying, Kistler developed the second mode (supercritical drying) during the 1930s.^58^ The aim is to eliminate the impact of the surface tension while operating at supercritical conditions of the interstitial solvent. Ideally, CO_2_ is used a lot because it has several advantages: (i) its critical temperature and pressure which are more moderate, (ii) its high chemical inertness, (iii) its non-flammability, (iv) its non-toxicity, and (v) its low cost. Furthermore, it is miscible with many common organic solvents,^59^ which facilitates an advantage of the drying process, after replacing the synthesis solvent with another miscible with CO_2_ such as alcohols to obtain alcogels.^60,61^

In the context of a series of research studies that we are leading dealing with porous nanomaterials, we were able to synthesize Ag/ZnO xerogels by an air drying process in a previous publication.^62^ The as-obtained xerogels exhibit a shrunken random micro-structure with limited porosity and a low surface area. Aiming to compare the advantages of aerogel materials, in our present research study, we investigate the effect of a supercritical CO_2_ drying process on the physical–chemical properties of ZnO materials. The supercritical drying process was chosen in order to replace the liquid in the prepared material with CO_2_ gas which isolates the solid component from the material without destroying the material’s delicate nanostructured pore network. To the best of our knowledge, the preparation of ZnO foam-structures *via* gelation of alginate by Zn^2+^ using supercritical CO_2_ drying technology has never been published before. It is in this context that this current study is being taken, to meet the objective of preparing ZnO foams by a novel, simple, low-cost and ecofriendly approach. Thus, we described the eco-design of Alg/Zn *via* alginate gelation by Zn^2+^ cations followed by supercritical CO_2_ drying and heat treatment of the ensuing aerogels which leads to ZnO foams. A comparative study of conventional drying *versus* supercritical drying was conducted to determine the influence of each mode of drying on the passage of the hybrid material (xerogels or aerogels) to the hard material (zinc oxide).

## 2. Experimental

### 2.1. Materials

Zn(NO_3_)_2_·6H_2_O (98%) and sodium alginate (SA, 99.99%) were purchased from Aldrich and used without any further purification. The water used in all experiments was deionized.

### 2.2. Preparation of ZnO nanomaterials

**Fig. 1:**
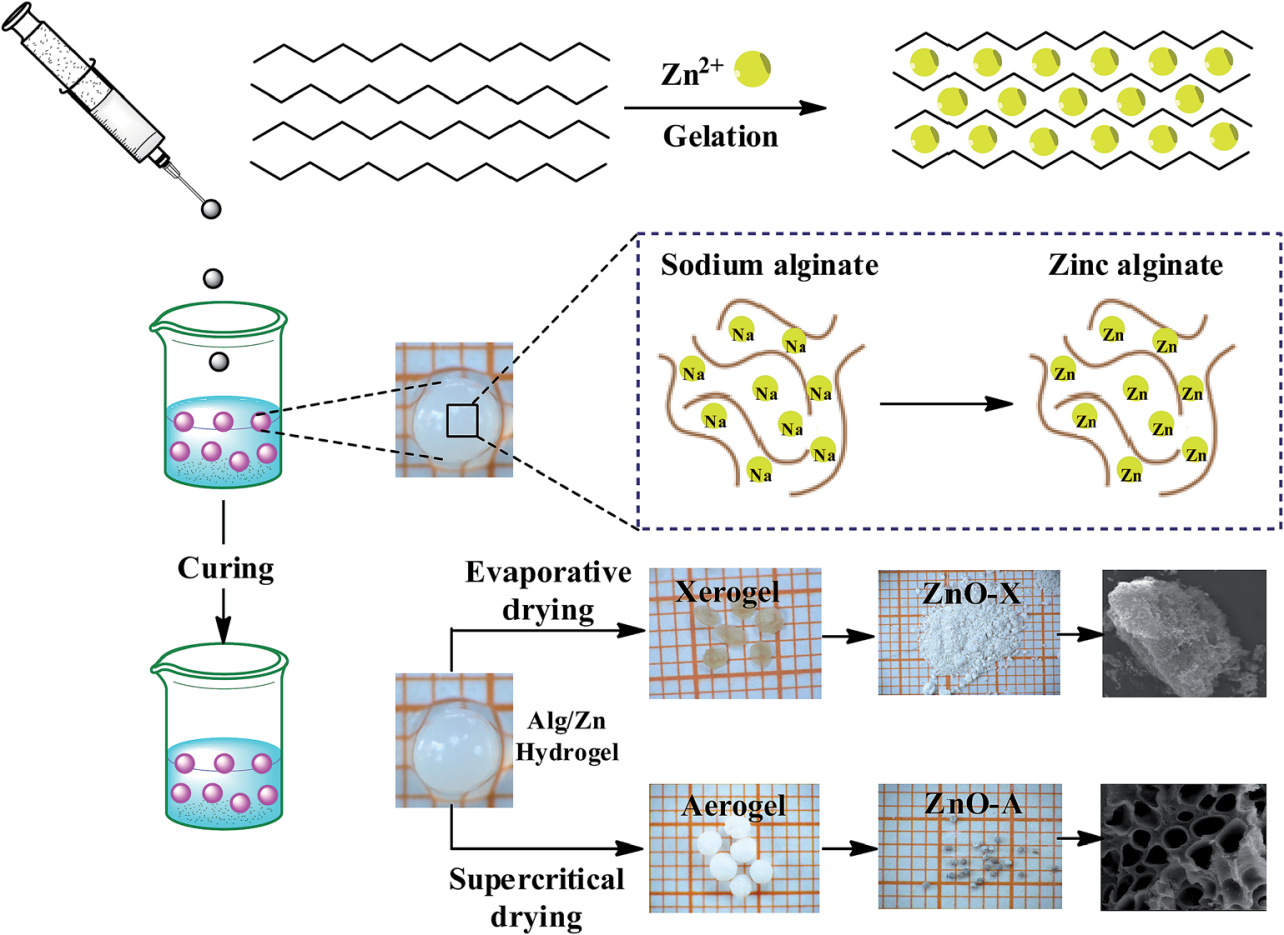
Schematic representation of the chemical route used for the synthesis of ZnO aerogel and xerogel nanomaterials.

The preparation of ZnO nanomaterials involves several steps, which are based on our previous work.^62^Fig. 1 presents the chemical route used for the synthesis of ZnO nanomaterials.

The first step consists of the preparation of the hydrogel beads by cross-linking reactions between the alginate solution and the Zn^2+^ aqueous solution. The shape and size of the hydrogel microspheres can be controlled by the method of bringing the two solutions into contact. Gelling of the alginate occurs when Zn^2+^ cations were cross-linked in the inter-chain binding between the biopolymer chains, affording a three-dimensional open network. This method leads to the spontaneous diffusion of a Zn^2+^ ion inside the alginate chains and formation of a three-dimensional network of well-shaped white microspheres, as shown in Fig. 1. In a typical experiment, 1 g of SA was dissolved in 100 mL of deionized water (18 MΩ) at room temperature, forming a viscous solution. A separate solution was prepared by dissolving an amount of Zn(NO_3_)_2_·6H_2_O in 100 mL of deionized water to obtain solutions with different final concentrations: 0.1, 0.2, and 0.3 M. Then, the SA gel (1%: w/w) was added dropwise at room temperature to the zinc(ii) precursor solution *via* a syringe with a 0.8 mm diameter needle and the mixture was constantly stirred for 2 hours. The obtained hydrogel beads (Alg/Zn) were processed *via* two different drying routes. The first one was a conventional air-drying method performed at room temperature to obtain xerogel beads (denoted Alg/Zn-X1, Alg/Zn-X2 and Alg/Zn-X3). The second method deployed was drying with supercritical CO_2_ to generate aerogel beads (denoted Alg/Zn-A1, Alg/Zn-A2 and Alg/Zn-A3). For this, hydrogel beads were first transformed into alcogels by putting the hydrogels in a succession of different ratios of water/absolute ethanol: 100/0, 95/5, 90/10, 80/20, 70/30, 60/40, 50/50, 40/60, 30/70, 20/80, 10/90 and 0/100. The alcogels were then placed in a supercritical reactor where liquid CO_2_ was injected under pressure and the mixture was heated to the critical temperature of CO_2_ (31.1 °C at 7.4 MPa). Both forms of the prepared materials (aerogels or xerogels) were calcined at a temperature estimated from the analysis of these materials using TGA. The calcined xerogel beads were coded ZnO-X1, ZnO-X2, and ZnO-X3, and the calcined aerogel beads were coded ZnO-A1, ZnO-A2 and ZnO-A3.

### 2.3. Characterization

The gel microspheres were dried under supercritical CO_2_ conditions (74 bar, 31.5 °C) using a PolaronE3100 Critical Point Dryer (Quorum Technologies). Thermo-gravimetric analysis (TGA) was conducted under air using TA Instrument Q500 apparatus, with a 10 °C min^−1^ ramp between 25 and 1000 °C. Fourier transform infrared (FT-IR) spectra of samples in KBr pellets were measured on a Bruker Vector 22 spectrometer. X-ray diffraction patterns of the uncalcined and calcined xerogel and aerogel spheres were recorded on a Bruker AXS D-8 diffractometer using Cu-Kα radiation in Bragg–Brentano geometry (*θ*–2*θ*). Scanning electron microscopy (SEM) micrographs were obtained using an FEI Quanta 200 microscope after carbon metallization. TEM micrographs were obtained on a Tecnai G2 microscope at 120 kV. Nitrogen adsorption/desorption isotherms were recorded using Micromeritics 3Flex apparatus at 77 K after outgassing the sample at 150 °C overnight. The surface areas were determined from the nitrogen adsorption/desorption isotherms, using the BET (Brunauer–Emmett–Teller) method. The pore size distributions were determined according to the BJH (Barrett–Joyner–Halenda) method. RTPL analysis was performed using a Horiba Yvon Jobin spectrophotometer.

## 3. Results and discussion

**Fig. 2:**
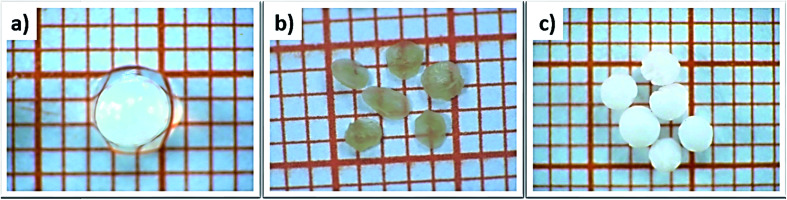
Digital images of (a) Alg/Zn hydrogels, (b) Alg/Zn xerogels and (c) Alg/Zn aerogels (grid square side is 1 mm).

The influence of the drying process on the macroscopic morphology of the microsphere samples is illustrated in Fig. 2. At the macroscopic scale it is clear that Alg/Zn xerogel beads shrunk to as much as 70% of their initial volume and lost their spherical form (Fig. 2b), while the Alg/Zn aerogel beads preserved the spherical shape of the Alg/Zn hydrogel beads, but their size was reduced to 50% of their original size (Fig. 2c). The evaporative drying has induced a dramatic shrinking and contraction of the Alg/Zn spheres resulting in the formation of a compact alginate with virtually no porous structure. In contrast, the supercritical drying brings about a limited volume shrinkage indicating that the Alg/Zn aerogel beads preserve the texture of the parent hydrogel form. The supercritical drying is crucial to form the aerogel scaffold *via* scattering of the nanometric polysaccharide fibrils which led to the preservation of the dispersion of the alginate biopolymer (Fig. 2c). It is important to point out that the most significant shrinkage was observed essentially during the dehydration of the hydrogel and is virtually negligible during the CO_2_ supercritical drying of the alcogel.

**Fig. 3:**
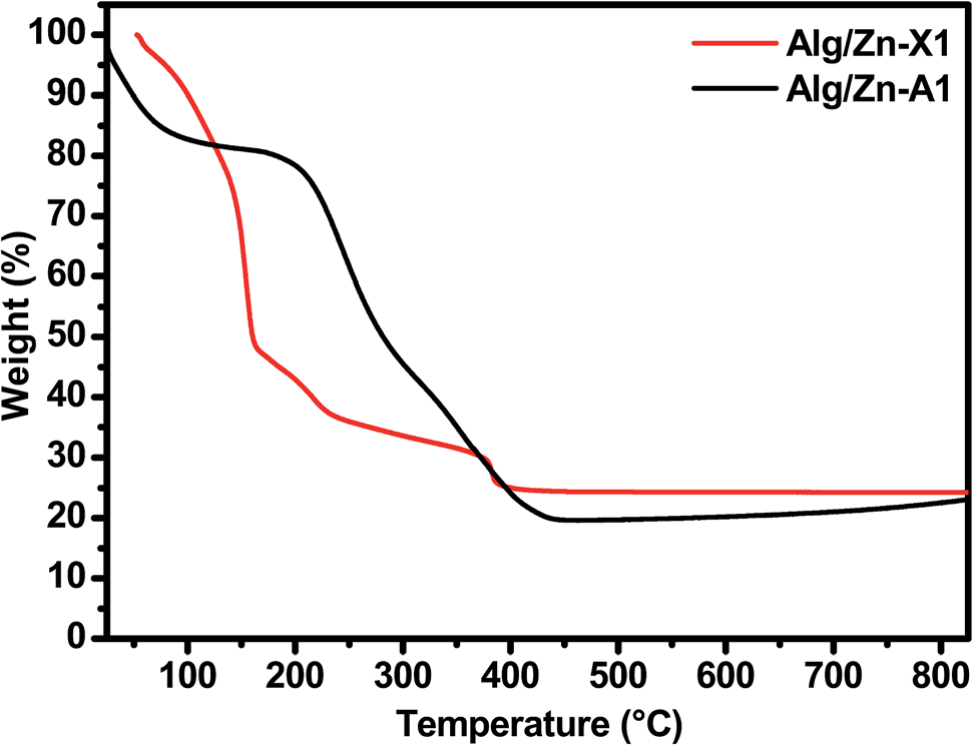
TGA curves of Alg/Zn-X1 and Alg/Zn-A1 samples.

Thereafter, a heat treatment of Alg/Zn xerogel and aerogel bead materials was performed to release the inorganic matrix and to obtain the zinc oxides ZnO-X1, ZnO-X2, ZnO-X3, ZnO-A1, ZnO-A2 and ZnO-A3. After that, the minimum calcination temperature necessary to remove organic residues was determined by thermo-gravimetric analysis; the details are described in the ESI (Fig. A1).† In the following section, we restrict ourselves to the results of the thermal treatment of Alg/Zn-X1 and Alg/Zn-A1 (Fig. 3). The thermal degradation of the Alg/Zn-X1 sample showed three weight losses. The first weight loss (11.66%) at 156 °C can be attributed to the evaporation of surface bonded water, the second weight loss of 63.77% observed at 218 °C can be assigned to the thermal degradation of alginate *via* decarboxylation,^61^ and the third weight loss (75.24%) around 385 °C can be attributed to full degradation of the alginate backbone. In the case of the Alg/Zn-X1 sample, we showed the first weight loss at 93 °C due to the removal of the alcohol residues remaining after CO_2_ supercritical drying, while the second weight loss (54.09 wt%) and third weight loss (80.49 wt%) which occurred at 254 and 383 °C respectively can be attributed to the thermal degradation of the alginate.^62^ Thus, the Alg/Zn aerogel and xerogel were calcined at 500 °C for 4 h under air wherein the Alg/Zn xerogel gave definite nanostructured zinc but this was not foamy in nature.

**Fig. 4:**
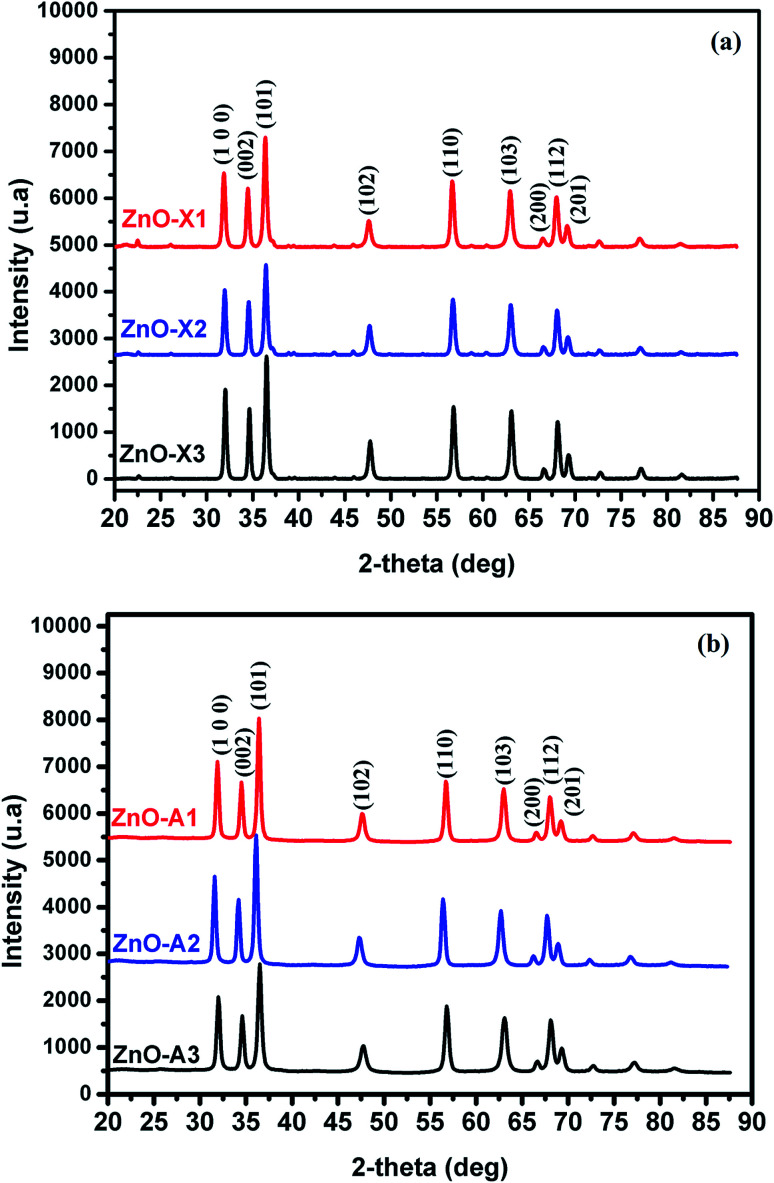
X-ray diffraction patterns of (a) ZnO-X1, ZnO-X2, and ZnO-X3 and (b) ZnO-A1, ZnO-A2 and ZnO-A3.

All samples prepared in this study have been characterized by X-ray diffraction. Firstly, we should note from this analysis that the Alg/Zn xerogel and aerogel beads are amorphous (Fig. A2†), thus no typical diffraction peaks were detected. Fig. 4 shows the X-ray diffraction patterns of ZnO-X1, ZnO-X2, ZnO-X3, ZnO-A1, ZnO-A2 and ZnO-A3. The ensuing zinc oxides exhibit hexagonal phases with wurtzite faces for all of the samples, according to the international database (PCDJ 001 036 054), and the main crystallographic planes are (100), (002), (101), (102), (110), (103), (200), (112) and (201). As can be seen in Fig. 4, the presence of intense and narrow shaped peaks indicates the formation of highly crystalline ZnO nanoparticles. The crystallite sizes calculated using the Debye–Scherrer formula are depicted in Table 1. These results revealed that the concentration of the Zn precursor significantly affected the crystallite size of the ZnO phase. In the cases of ZnO-X1, ZnO-X2 and ZnO-X3, the crystallite size was increased from 19.7 to 22 nm. The dependence of the crystallite size on the Zn^2+^ concentration could be due to the Ostwald ripening phenomenon. At higher concentrations, a high-density network of small nanoparticles seems to be formed when compared to lower concentrations, in which the formed small nanoparticles were relatively far from each other. Furthermore, the crystallite size of ZnO samples was also affected by the method of drying. As can be seen in Table 1, supercritical drying of ZnO samples was observed to give the smallest crystallite size compared to the case of using conventional drying. These results can be explained by the elimination of capillary force among particles during supercritical drying, which can inhibit particle agglomeration.^63^

**Table 1:** Lattice parameters, crystallite sizes and cell volumes of all samples

Samples	ZnO-X1	ZnO-X2	ZnO-X3	ZnO-A1	ZnO-A2	ZnO-A3
Lattice parameter (nm)	3.2422	3.2394	3.2392	3.2391	3.2444	3.2440
	5.1962	5.1891	5.1899	5.1937	5.2022	5.2000
Cell volume (Å^3^)	47.300	47.150	47.156	47.190	47.420	47.390
Crystallite size (nm)	19.7	22.8	22	17.8	20.2	21.4

**Fig. 5:**
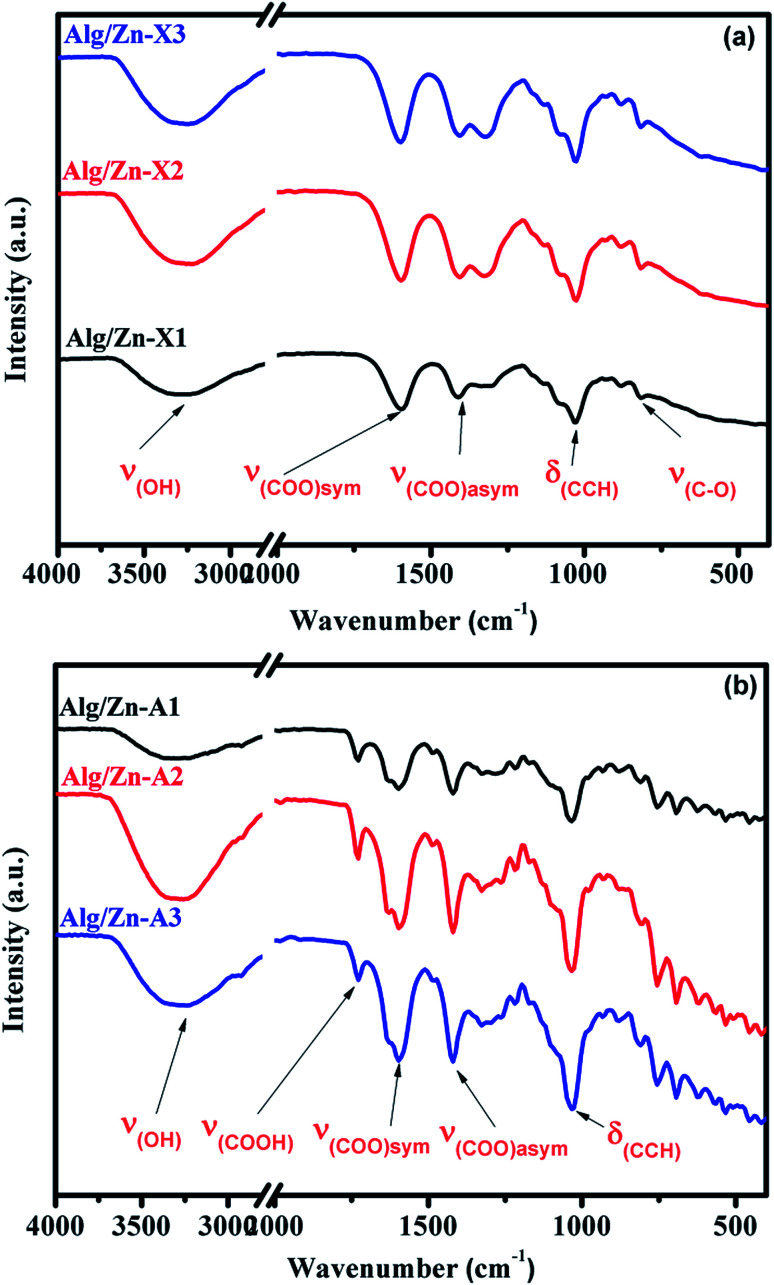
FTIR spectra of Alg/Zn xerogels and aerogels at different Zn^2+^ concentrations.

In the ionotropic gelling of alginate, zinc was chelated by two carboxylic groups of two polymer chains *via* electrostatic bridges which led to the formation of a stiffened gel. To understand the influence of this complexation on the vibrational bands of alginate, both alginate gel and cross-linked Alg/Zn xerogel and aerogel beads prepared using three different concentrations of the zinc source were analyzed using FT-IR spectroscopy. Fig. 5 shows the FTIR spectra of the alginate gel, and the Alg/Zn xerogel and aerogel beads with different concentrations. According to these figures, all analyzed samples exhibited the same vibrational modes and the same characteristic bands (Table 2). On the other hand, no characteristic vibrational bands ascribed to the crystalline phase of zinc oxide were observed in the FTIR spectra of both Alg/Zn xerogel and aerogel beads, suggesting that these samples are hybrid materials formed by amorphous zinc oxide. These findings are in agreement with those obtained in our previous study.^62^

**Table 2:** Attribution of the main vibrational modes of Alg/Zn xerogel and aerogel materials

Sample	(O–H)	(COO^−^)_asym_	(COO^−^)_sym_	(C–O)
Sodium alginate	3244	1596	1404	1023
Alg/Zn-X1	3278	1590	1415	1026
Alg/Zn-A1	3269	1594	1422	1030

**Fig. 6:**
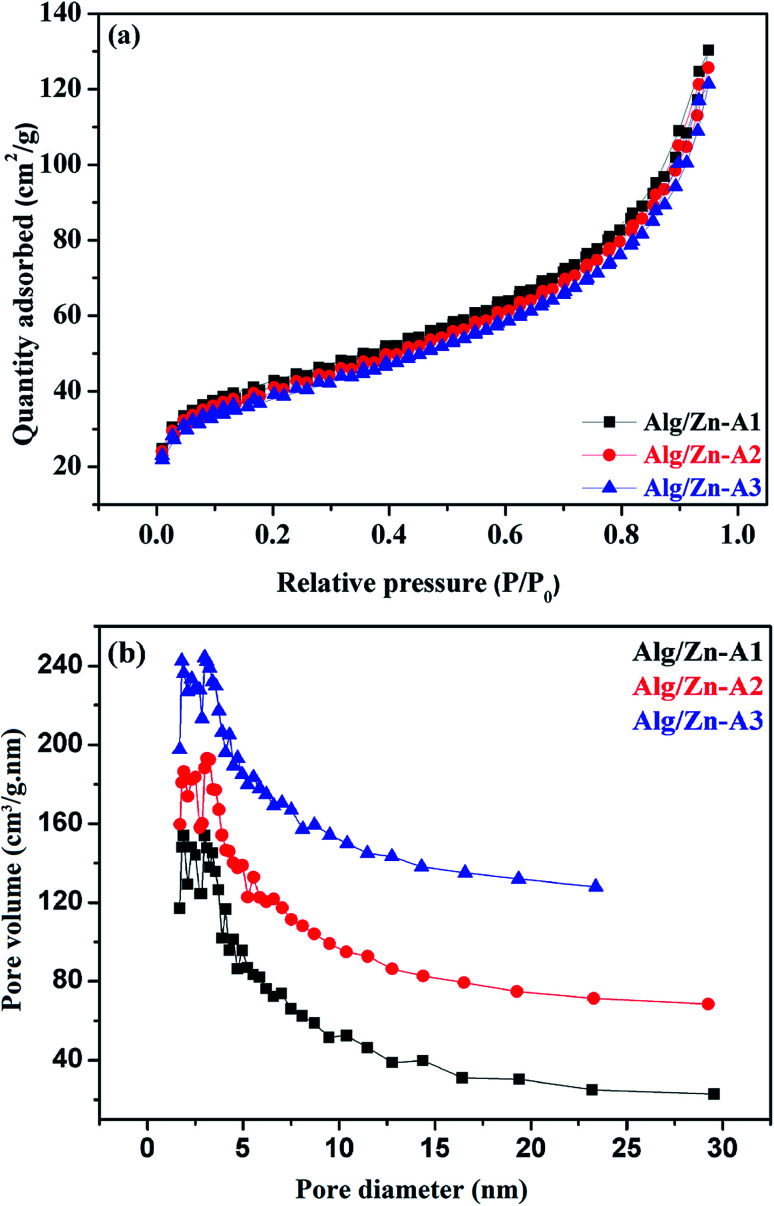
(a) N_2_ adsorption–desorption isotherms and (b) pore size distributions of Alg/Zn-A1, Alg/Zn-A2 and Alg/Zn-A3.

The N_2_ adsorption–desorption technique has been applied to determine the textural properties of all of the prepared samples. First of all, it should be noted that Alg/Zn xerogel beads are considered as non-porous materials since the corresponding N_2_ adsorption–desorption isotherms were difficult to collect.^64,65^ In this case, the most appropriate technique to evaluate the textural properties of the Alg/Zn-X samples is intrusion mercury porosimetry. Firstly, the N_2_ adsorption–desorption isotherms of the three Alg/Zn aerogel beads prepared using three different concentrations of zinc solution are reported in Fig. 6a. As can be seen, all of the recorded isotherms are intermediate between type II and type IV with a distinct H2-type hysteresis loop according to the IUPAC classification,^65^ indicating the presence of a significant mesoporosity with an interconnected network and a high outer surface area. Similarly, the isotherms of the solid obtained by calcination of both xerogel and aerogel beads at 500 °C are also intermediate between type II and type IV.

The surface areas of all of the samples were determined by nitrogen adsorption (BET method). Based on the results obtained, the surface areas of the three materials Alg/Zn-A1, Alg/Zn-A2 and Alg/Zn-A3 were 143, 137 and 131 m^2^ g^−1^ respectively, which indicates that the surface area of the aerogel microspheres is slightly affected by the concentration of the zinc precursor and also the method of drying. Furthermore, the pore size distribution of Alg/Zn-A1, Alg/Zn-A2 and Alg/Zn-A3 presents narrower mesopore distributions centered around 6.938, 7.052 and 7.171 nm, respectively (Fig. 6b), indicating that this sample contains ordered mesopores as mentioned above. Such a result was expected since supercritical drying is expected to enable drying without leading to the collapse of the structure, affording an aerogel with a high surface area.

However, it can be observed that the calcined aerogels present a high surface area compared to the xerogels due to less pore shrinkage. The surface areas of ZnO-X1, ZnO-X2, and ZnO-X3 are 9.45, 13.04, and 11.94 m^2^ g^−1^, respectively, while the surface areas of ZnO-A1, ZnO-A2, and ZnO-A3 are 22.94, 16.62, and 14.21 m^2^ g^−1^, respectively. These results indicate that the surface area values depend on the concentration of the precursor used and also on the drying process adopted for the microsphere preparation. In comparison with our previous report,^62^ herein we report that the concentration of zinc used for the gel bead preparation significantly affected the textural properties of the produced zinc oxide since the surface area of the prepared zinc oxide increases when increasing the concentration of the zinc precursor. Also, it was demonstrated that the surface area of the zinc oxide nanomaterial not only depends on the zinc concentration but also on the adopted drying process since the surface area of the produced ZnO nanomaterial was slightly improved. Thus, the present study introduces a moderate approach for the synthesis of zinc oxide with improved textural properties.

**Fig. 7:**
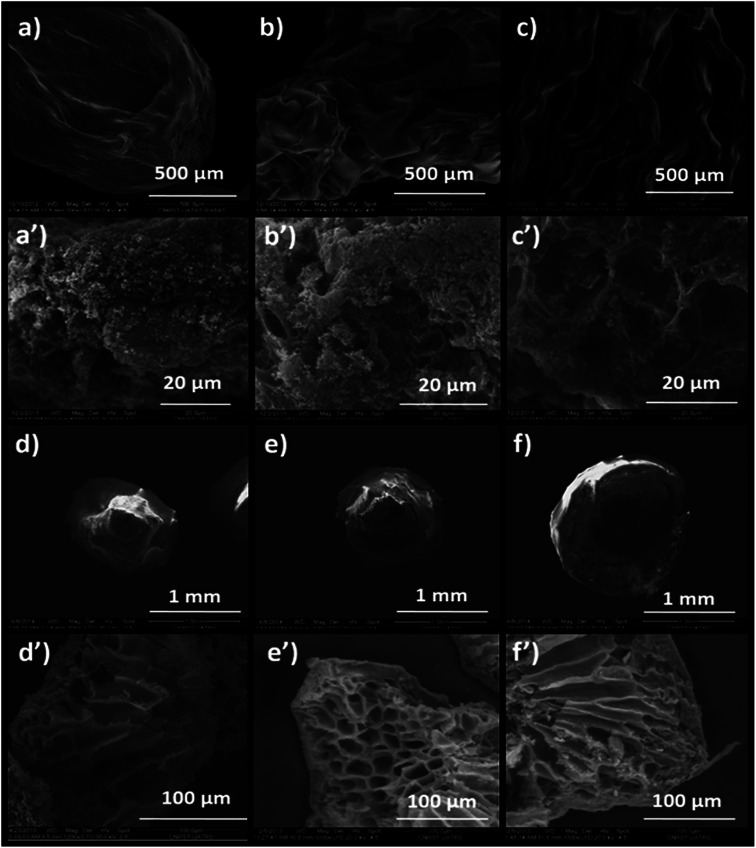
SEM images of: (a) Alg/Zn-X1, (b) Alg/Zn-X2, (c) Alg/Zn-X3, (a′) ZnO-X1, (b′) ZnO-X2, (c′) ZnO-X3, (d) Alg/Zn-A1, (e) Alg/Zn-A2, (f) Alg/Zn-A3, (d′) ZnO-A1, (e′) ZnO-A2 and (f′) ZnO-A3.

The influence of the drying mode on the morphological properties of the as-prepared materials was investigated by SEM. The SEM images in Fig. 7a–c present the morphologies of Alg/Zn-X1, Alg/Zn-X2 and Alg/Zn-X3. Generally, a porous surface with roughened rib structures was observed for all xerogel samples. Furthermore, observation at high magnification revealed that when the zinc concentration increases, more visible folding was observed, and these folds become thicker at higher concentrations of zinc solution. In the case of Alg/Zn-A1, Alg/Zn-A2 and Alg/Zn-A3 (Fig. 7d–f), it is clearly observed that the drying mode had an obvious influence on the morphology of the prepared beads. Supercritical drying plays a very important role in the conservation of the bead morphology. As shown in Fig. 7d–f, the supercritically-dried Alg/Zn beads retain the spherical shape of the hydrogel but the size of the aerogel spheres is nearly half of the size of the parent hydrogel spheres. This drying process preserves the spherical-shaped morphology of the alcogel and avoids the pore collapse phenomenon *via* the reduction of the capillary tension between polysaccharide chains. It should be noted that the external diameter of the alcogel beads was reduced from 3 to 1.4 mm after supercritical drying. On the other hand, the scanning electron micrographs of ZnO-A1, ZnO-A2 and ZnO-A3 samples are shown in Fig. 7d′–f′. It is obvious that ZnO nanoparticles obtained from supercritically-dried aerogels exhibited a highly porous structure consisting of a hollow tubular network. Indeed, the supercritical drying technique was effective in preserving the network structure and preventing the shrinkage or collapse of mesopores, resulting in the formation of a well-defined porous structure. However, as can be seen in Fig. 7a′–c′, the ZnO-X1, ZnO-X2 and ZnO-X3 samples are porous yet contain a partially closed-cell structure. Furthermore, the observation of cross-sections of the microspheres indicates that the core of the bead is totally occupied by a radial pattern of channels, lined by a macroporous alginate matrix. Besides, the examination of SEM images reveals that the ZnO samples tend to become more porous when the concentration of the Zn^2+^ was increased from 0.1 to 0.3 M. Additionally, the mesopores of the calcined aerogels became more accentuated and strongly interconnected when compared with the calcined xerogels.

**Fig. 8:**
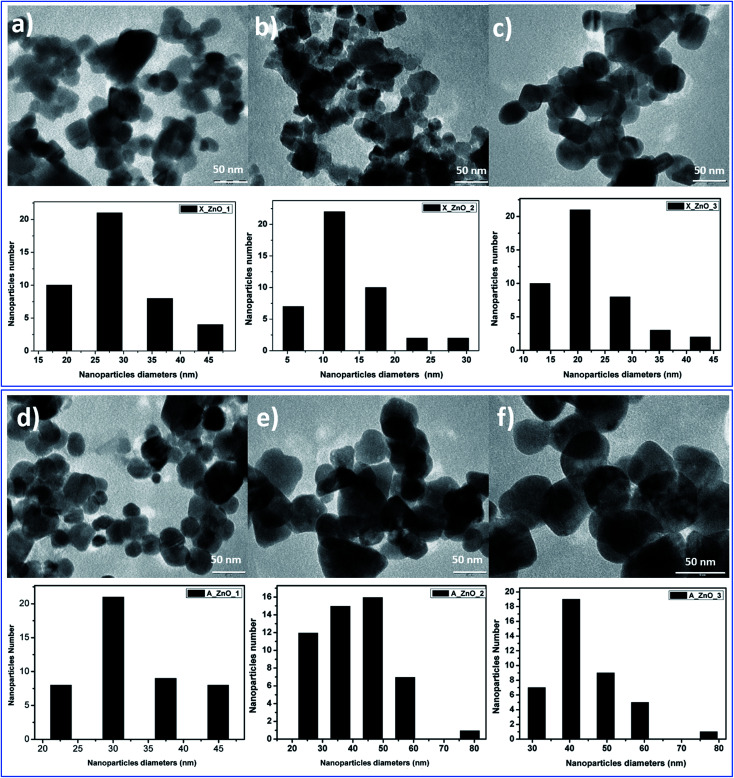
TEM images of ZnO nanoparticles obtained from fine grinding of ZnO foams: (a) ZnO-X1, (b) ZnO-X2, (c) ZnO-X3, (d) ZnO-A1, (e) ZnO-A2 and (f) ZnO-A3.

The obtained ZnO nanocrystals were further characterized by TEM. Fig. 8 shows the TEM images of the ZnO nanocrystals obtained by thermal decomposition of both Alg/Zn xerogels and aerogels. As can be seen in Fig. 8, at low concentrations of zinc solution, a quasi-spherical shape was obtained, whereas at relatively higher concentrations, regular spherical-shaped nanoparticles were observed, supporting the idea of the Ostwald ripening phenomenon. Indeed, the nanoparticle size is bigger than the crystallite size, suggesting that particles can be formed by one to several crystallites. Furthermore, the nanoparticle size distribution revealed that the nanoparticle size ranges between 13.6 and 31.37 nm for ZnO-X1 and ZnO-X3 respectively, and between 30.36 and 44.2 nm for ZnO-A1 and ZnO-A3 respectively. It is clearly observed that the mean particle size seems to be influenced by the kind of drying process. Conventional drying results in the formation of smaller particles with a more uniform distribution than supercritical drying. This can be due to the different binding capacities arising from zinc alginate and zinc nitrate. Moreover, this difference in particle size could also be due to a large difference in growth times between the conventionally- and supercritically-dried gel beads.

In contrast to our previous study^62^ where 20 nm quasi-spherical ZnO nanoparticles were obtained, the spherical particles in Fig. 8 are much larger, probably due to the chosen zinc concentration and the adopted drying method. Indeed, it can be seen that there is a significant increase in the particle size with increasing zinc concentration for both conventionally- and supercritically-dried samples calcined at the same temperature.

**Fig. 9:**
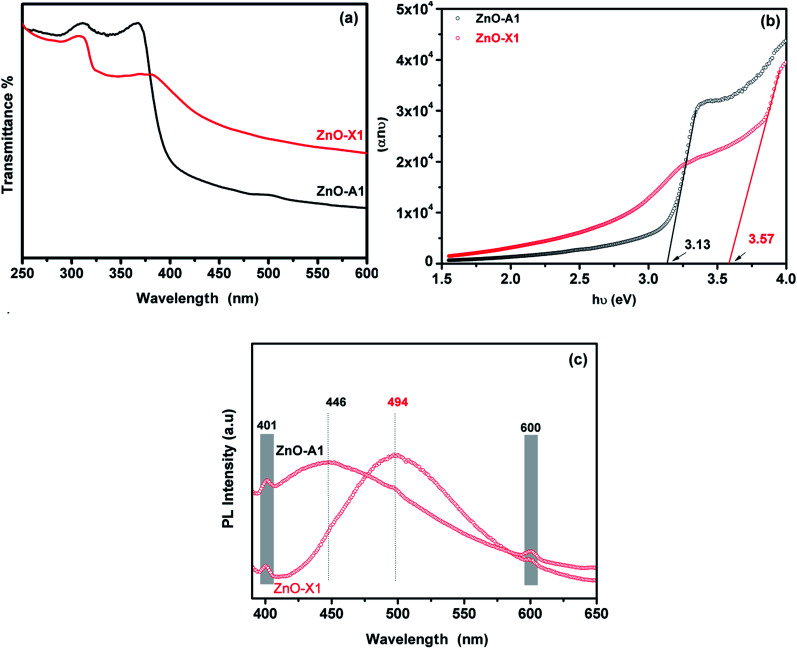
(a) UV-Vis absorption spectra, (b) Tauc plot and (c) RTPL emission analysis of ZnO-X1 and ZnO-A1 nanomaterials.

The UV-Visible absorption spectra of the as-synthesized ZnO-X1 and ZnO-A1 are shown in Fig. 9a. As can be seen, all of the analyzed samples exhibited two excitonic absorption bands in the range of 250–450 nm. The ZnO-A1 nanomaterial showed two strong adsorption bands at around 310 and 365 nm while the ZnO-A1 sample exhibited typical adsorption bands at approximately 307 and 381 nm. It should be noted that the same pattern was observed for all of the prepared ZnO samples. For the ZnO-A1 nanomaterial, the absorption band located at 365 nm was blue shifted by about 16 nm compared to that for the ZnO-X1 sample, which appears at 381 nm. Such displacement was probably due to the pronounced quantum confinement effect in the ZnO nanocrystallites.^66^ The band gaps (*E*_g_) of ZnO-X1 and ZnO-A1 are estimated using a Tauc plot, extrapolating the linear portion of the plot of (*αhν*)^2^*vs.* photon energy (*hν*) (Fig. 9b). The typical linear fittings of ZnO-X1 and ZnO-A1 are shown in Fig. 9b. The *E*_g_ values of ZnO-X1 and ZnO-A1 were found to be 3.57 and 3.13 eV, respectively. Furthermore, RTPL emission analysis of both ZnO-X1 and ZnO-A1 was performed. As shown in Fig. 9c, two bands at 401 (blue emission) and 600 nm (red emission), due to zinc vacancy related defects,^67,68^ are observed for both materials. In addition, broad bands in the blue-green region are observed at 494 and 446 nm for ZnO-X1 and ZnO-A1, respectively, and these can be associated with the oxygen vacancy related defects.^69^

## 4. Conclusions

In summary, sodium alginate gelation has been demonstrated to be an efficient method for the green synthesis of nanostructured ZnO materials. The drying process and the concentration of the zinc solution were found to be crucial to minimize shrinkage and to control the morphology and the size of the Alg/Zn beads. For comparison, Alg/Zn beads were also dried using a conventional evaporation process of the interstitial solvent. We investigated the surface area, pore volume, pore diameter and morphology of the Alg/Zn beads. ZnO nanocrystallites with sizes in the range of 17.8–22.8 nm were obtained by thermal treatment of the zinc–alginate beads previously dried by either evaporative or supercritical drying. XRD, SEM and TEM studies confirmed the nanostructures of the prepared ZnO nanoparticles. The prepared ZnO nanoparticles exhibit highly porous structures consisting of hollow tubular networks. From the TEM analysis, it was found that ZnO nanoparticles are of spherical shapes with average sizes of 13.6–44.02 nm. The UV-Visible spectra show blue shift absorptions at 310 and 365 nm for the ZnO-X1 sample and 307 and 381 nm for the ZnO-A1 sample. It was found that the band gap energies were 3.57 and 3.13 eV for ZnO-X1 and ZnO-A1, respectively. The RTPL analysis confirmed the ZnO structure defects. The proposed synthetic route for the formation of ZnO nanoparticles was simple, cheap, efficient, and free of pollution, which makes it very suitable for scaled-up production.

## Conflicts of interest

There are no conflicts to declare.

## Supplementary Material

RA-008-C8RA02129E-s001
