# Supplementary material for: Supercritical CO2 drying of alginate/zinc hydrogels: a green and facile route to prepare ZnO foam structures and ZnO nanoparticles
Source: RSC Adv. 2018 Jun 6;8(37):20737–47. doi: 10.1039/c8ra02129e (PMC9080889; doi:10.1039/c8ra02129e)
Supplement: RA-008-C8RA02129E-s001 [file RA-008-C8RA02129E-s001.pdf]

Electronic Supplementary Material (ESI) for RSC Advances.
This journal is © The Royal Society of Chemistry 2018

Supporting information

Supercritical CO_2 drying of Alginate/zinc hydrogel: Green and Facile route to Prepare ZnO Foamy-Structure and ZnO Nanoparticles

Hicham Abou Oualid,[a,b] Othmane Amadine,[b] Younes Essamlali,[b] Karim Dânoun,[b] Mohamed Zahouily[a,b*]

[a]*Laboratoire de Matériaux, Catalyse & Valorisation des Ressources Naturelles, URAC 24, Faculté des Sciences et Techniques, Université Hassan II, Casablanca B.P. 146, 20650, Morocco.*
[b]*MAScIR Foundation, VARENA Center, Rabat Design, Rue Mohamed El Jazouli, Madinat Al Irfane 10100 Rabat, Morocco.*

* Correspondence: m.zahouily@mascir.com ; Tel. +212661416359

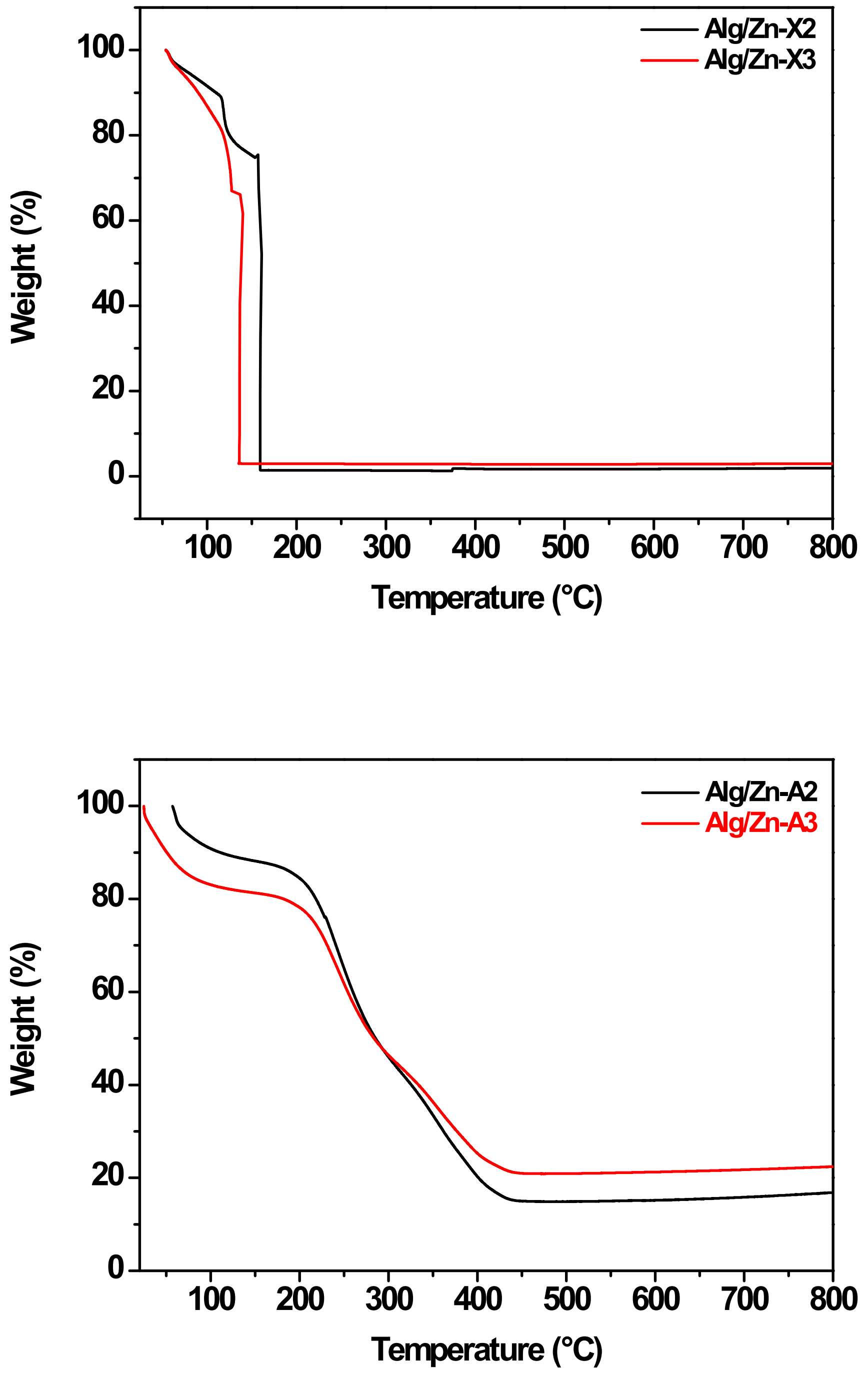

Fig. A1. TGA curves of the Alg/Zn-X2, Alg/Zn-X3, Alg/Zn-A2 and Alg/Zn-A3 materials.

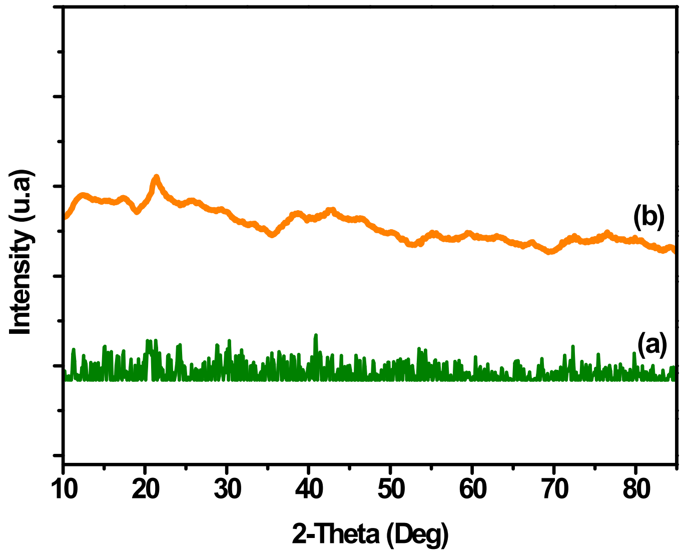

Fig. A2. XDR pattern of (a) Alg/Zn-X1 and (b) Alg/Zn-A1 dried on air and CO_2 supercritical conditions respectively.

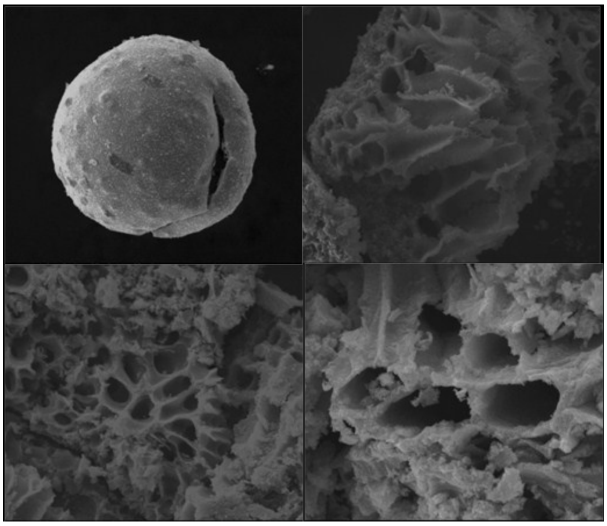

Fig. A3. SEM images of ZnO-A1 foams.

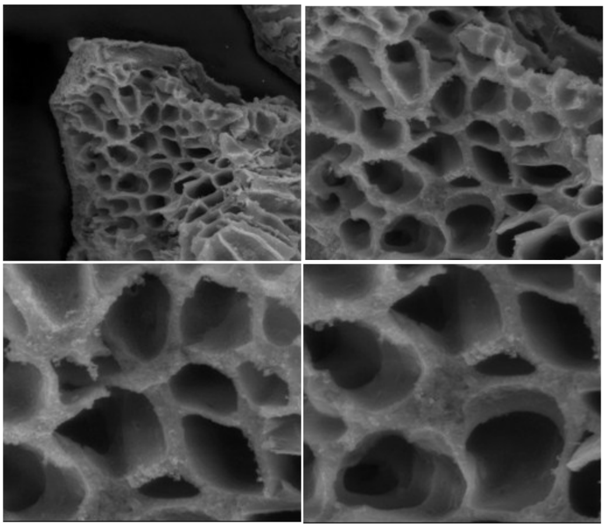

Fig. A4. SEM images of ZnO-A2 foams.

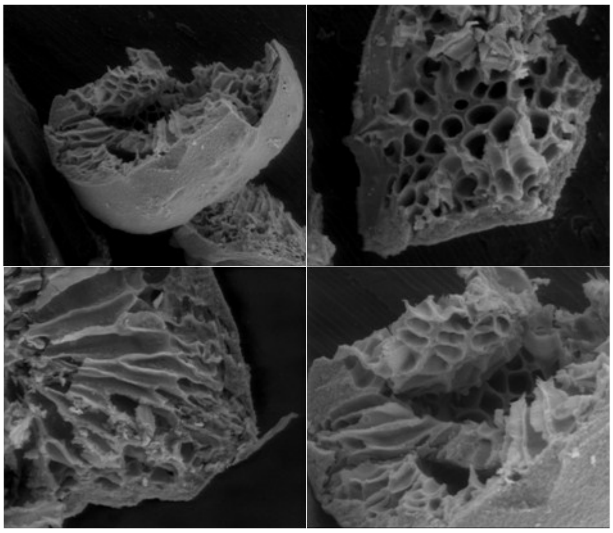

Fig. A5. SEM images of ZnO_A3 foams.
